# Metacognitive Performance on Memory and Visuospatial Tasks in Functional Cognitive Disorder

**DOI:** 10.3390/brainsci11101368

**Published:** 2021-10-19

**Authors:** Catherine Pennington, Harriet Ball, Marta Swirski, Margaret Newson, Elizabeth Coulthard

**Affiliations:** 1 Edinburgh Dementia Prevention, Centre for Clinical Brain Sciences, The University of Edinburgh, Edinburgh EH16 4SB, UK; 2 ReMemBr Group, Bristol Medical School, University of Bristol, Bristol BS10 5NB, UK; harriet.ball@bristol.ac.uk (H.B.); marta.swirski@bristol.ac.uk (M.S.); Margaret.newson@nbt.nhs.uk (M.N.); Elizabeth.coulthard@bristol.ac.uk (E.C.)

**Keywords:** metacognition, insight, anosognosia, functional cognitive disorder, mild cognitive impairment, neurodegeneration

## Abstract

Functional Cognitive Disorder (FCD) is a common diagnosis at the memory clinic. FCD is characterised by significant self-reported cognitive symptoms in the absence of external evidence of cognitive dysfunction. A potential explanation for this is a deficit in metacognition, the process by which we internally judge our own abilities. Here we investigated differences in accuracy, confidence, and metacognition between people with FCD (N = 20), neurodegenerative mild cognitive impairment (nMCI; N = 14), and healthy controls (N = 23). The groups were assessed on forced choice memory and perceptual tasks, with trial by trial confidence ratings. FCD and nMCI participants showed lower accuracy on the memory task (means FCD 63.65%, nMCI 63.96%, HC 71.22%), with a significant difference between the FCD and HC groups after controlling for age and sex. There were no between-group differences in memory task confidence (means FCD 3.19, nMCI 3.59, HC 3.71). The FCD group showed greater confidence when longer time was allowed on the memory task. No between group differences in perceptual task accuracy (means FCD 63.97%, nMCI 64.50%, FCD 65.86%) or confidence (means FCD 3.71, nMCI 3.43, HC 3.88) were found. No differences in metacognitive efficacy emerged between the groups, either on the memory or perceptual task (Memory Meta-d’/d’:FCD 0.63, nMCI 0.94 HC 0.85; Perceptual Meta-d’,d’: FCD 0.50, nMCI 0.51, HC 0.72). Participants showed greater metacognitive efficacy on the memory task compared to the perceptual task. The difficulties experienced by people with FCD do not appear to be due to metacognitive deficits. Their performance was similar to people with nMCI over aspects of the memory tasks, which suggests that the primary issue may lie with memory encoding or retrieval, rather than with their judgement of performance accuracy.

## 1. Introduction 

Metacognition refers to our knowledge of our own abilities and attributes; how good you are at certain tasks and where you may struggle. We use metacognition everyday—when estimating if you need to write down an appointment or shopping list, or when deciding to delegate a DIY task to a professional [1]. People with dementia often experience altered metacognition—this is also commonly called anosognosia, reduced insight, or altered symptom awareness [2]. Affected individuals may deny having memory problems, or continue to operate household appliances and drive despite not being safe to do so. There is emerging evidence that metacognition may be affected even in the early stages of prodromal neurodegeneration. Those with mild cognitive impairment (MCI) and impaired metacognition are more likely to progress to future dementia than those with intact awareness [3]. How metacognition for different cognitive domains changes over the course of neurodegeneration is unclear, and is an understudied topic.

There is an increasing push towards early diagnosis of dementia, and a focus on identifying those with prodromal neurodegeneration. A particular challenge in the cognitive clinic is making an accurate, aetiologically-based diagnosis for those with mild or variable cognitive symptoms. Potential causes of self-reported symptoms include prodromal neurodegeneration, the side effects of cognitive toxins, primary psychiatric diagnoses, and Functional Cognitive Disorder (FCD) [4]. FCD can be defined as a presentation with persistent, distressing subjective cognitive symptoms with demonstrable internal inconsistency—manifesting as either significant variability in ability, or an incongruence between self-reported difficulties and performance in everyday life or neuropsychological testing [5]. For a diagnosis of FCD there should be no evidence of a neurodegenerative, psychiatric, toxic, or systemic cause for the cognitive symptoms. FCD is an emerging diagnostic entity, and as such, the diagnostic criteria are in a state of evolution. Functional disorders are extremely common and can manifest in a wide array of bodily systems, with Functional Neurological Disorder being frequently diagnosed in the neurology clinic [6]. The underlying root cause for FCD is not yet clear, although in some patients a clear link with stressful life events or excessive concern about future dementia may be present. Why a gulf between perceived and real-world performance emerges is unexplained. One potential reason for the disconnect between perceived and actual cognitive functioning in FCD is a deficit in metacognition. In this instance, unlike the lack of symptom awareness seen in neurodegeneration, individuals over-estimate the severity of their cognitive symptoms. Theoretically, therefore, a measure of metacognitive efficacy may be able to distinguish people with MCI due to early neurodegeneration from those with FCD.

There remain many unknowns regarding human metacognition. It is uncertain as to whether perception of our cognitive abilities is specific to different cognitive domains such as memory (domain specific metacognition), or if we maintain an overarching view of our functioning at a global cognitive level (domain general metacognition) [7] or both. Task difficulty and individual performance will also influence one’s estimation of ability and accuracy. One approach to evaluating metacognitive accuracy is to capture trial by trial performance and confidence on a single domain cognitive task of varying difficulty. An individual who correctly evaluates their performance demonstrates highly effective metacognition, whereas one who under or over estimates their ability can be said to have impaired metacognition. Signal detection theory has been applied to the problem of dissecting metacognitive efficacy from performance [8]. The meta-d’/d’ ratio returns a quantitative measure of how well confidence levels differentiate correct and incorrect responses.

We hypothesised that people with FCD would show impaired mnestic metacognition, with a tendency to under-rate their performance, whilst those with neurodegenerative MCI (nMCI) would over-rate their abilities. This hypothesis is based on the inconsistency between self-reported cognitive functioning and performance in FCD [4,9]. This is similar to the discrepancies seen in other functional neurological disorders—people with functional tremor over-report the presence of tremor, as measured by wearable actigraphy [10]. People with functional weakness may report lower limb weakness that is inconsistent with their ability to walk [11]. Therefore, we hypothesised that when directly assessed, people with FCD would under-rate their own cognitive performance.

In contrast to this, people with dementia very often show a lack of insight into their memory and thinking symptoms [12]. Altered insight has also been demonstrated in people with MCI due to neurodegeneration, and may be an early indicator that the individual is at high risk of progressing to dementia [13]. This led to the second part of our hypothesis, namely that those with nMCI would show an excess of confidence in their performance.

FCD and nMCI were compared with a healthy control group with metacognitive efficacy, confidence, and performance accuracy evaluated across memory and perceptual forced choice tasks.

## 2. Materials and Methods

### 2.1. Participant Selection

Participants with FCD or nMCI were recruited from a specialist NHS tertiary referral cognitive disorders clinic. Diagnoses were made by a cognitive neurologist, following clinical assessment, neuroimaging, and neuropsychological assessment. All diagnoses were reviewed at multidisciplinary meeting involving three cognitive neurologists, a consultant neuropsychologist, and specialist nurse. All except one participant were clinically assessed on at least two occasions. In addition, one participant with nMCI was recruited from Join Dementia Research; they were diagnosed at a different NHS cognitive clinic and their clinical assessment reviewed by a consultant cognitive neurologist (C.P.) prior to participation in the study.

A diagnosis of FCD was made based on the presence of persistent, severe self-reported cognitive symptoms, with a marked discrepancy between symptoms and observed or reported good everyday cognitive functioning. Additionally, no evidence of an alternative diagnosis was found, and there was no evidence of progressive deterioration.

nMCI was diagnosed in patients with mildly impaired cognition on neuropsychological assessment, with preserved everyday abilities and evidence of underlying neurodegeneration (based on neuroimaging findings and a progressive decline over time).

Age-appropriate healthy controls (HC) were recruited from a local database of volunteers for cognitive research, and from Join Dementia Research (https://www.joindementiaresearch.nihr.ac.uk/, accessed on 1 November 2010). All controls were self-described as being cognitively healthy.

For all participant groups, those with major psychiatric diagnoses, toxic or metabolic causes of cognitive dysfunction, or significant systemic disease likely to adversely affect cognition were excluded. Those with mild low mood or anxiety, and those using low levels of potentially psychoactive medication were not excluded where the cognitive neurology team felt these were unlikely to impact on cognition. This was a deliberate strategy, as such scenarios are extremely common in the general population, and we wished to evaluate groups broadly representative of a ‘real world’ scenario.

### 2.2. Assessment Procedure

Participants completed demographic questionnaires, neuropsychological assessment and metacognitive evaluation. Neuropsychological battery included the Montreal Cognitive Assessment (MoCA), Test of Premorbid Functioning (UK version), the Prospective and Retrospective Memory Questionnaire, Hopkins Verbal Learning Task, Trails A and B, and the Minnesota Multiphasic Personality Inventory (results reported elsewhere [14,15].

Metacognition was assessed with two alternate forced choice tasks, described in detail in [16]. In brief, during the memory task, participants were shown 50 English words on the screen simultaneously, and instructed to memorize as many as possible. Study time was 30, 60, or 90 s. Participants were alerted when 10 s remained of the study time. Following the study time, a series of 2-alternative forced choice new/old judgements were completed. Two words were presented, 1 of which had previously appeared in the study list. Participants were asked to select the word previously presented. Four blocks of 50 trials were undertaken (1 block with 30 s study time, 2 blocks with 60 s study time, 1 block with 90 s study time). Following each trial, a screen with a sliding scale of confidence was presented and participants were instructed to rate their confidence that their response was correct (scale from 1.00, low confidence to 6.00, high confidence). Performance accuracy on the task was resulted as percentage correct over each block of study time. Confidence was reported as the mean rating given over each block of study time.

During the perceptual task, participants viewed a screen displaying two circles containing a variable number of dots (both circles and dots were white on a black background for maximum contrast). Participants were asked to select the circle they estimated to contain the most dots. The difference in dot number between the circles was staircased to ensure that participant performance was maintained at a consistent level (see [8,16] for details). Following two consecutive correct responses, the difference in dot number was reduced by one dot; following one incorrect response, the different in dot number was increased by one dot. The aim was to establish a consistent performance level for the perceptual task between different participants. Participants rated their confidence after each trial (in the same way as for the memory task). Each participant completed 8 blocks of 25 trials. The results of the perceptual task were given as the percentage correct over all trials, and the mean confidence rating by a participant over all trials.

Practice trials were provided prior to both the memory and perceptual tasks.

Subjective confidence ratings are influenced by task difficulty and performance. In order to derive an unbiased measure of metacognition, we calculated metacognitive efficiency. In this context this represents the participant’s ability to determine whether their response was correct or not. Metacognitive efficiency is reported as meta-d’/d’, where meta-d’ is a measure of type 2 sensitivity (ability to distinguish correct from incorrect responses), using the same units as type 1 sensitivity (d’; ability to distinguish stimulus alternatives) [17]. Meta-d’/d’ is computed using a signal detection theory model, as described in [8]. This provides a measure of metacognition that is independent of task performance, difficulty, and confidence. Under ideal performance conditions, meta-d’/d’ is 1 (i.e., one’s ability to distinguish between the choices is equal to ability to decide if one’s choice is right or wrong), whereas a result below 1 indicates sub-optimal metacognition. The MATLAB code used to fit meta-d’ to individual data is available at: http://www.columbia.edu/~bsm2105/type2sdt (accessed on 1 November 2010).

The Domain General Index (DGI) was derived by subtracting log (Meta-d’/d’)Memory from log (Meta-d’/d’)Perceptual. The DGI can be used to evaluate differences between perceptual and mnestic metacognitive efficacy. If no differences are present, the DGI will be 0, whereas if perceptual metacognition is superior to mnestic, the DGI will be positive, and if mnestic metacognition is better, the DGI will be negative. Seeking differences in metacognitive efficacy across different cognitive domains will further explore whether metacognition is domain specific or domain general.

### 2.3. Statistical Analysis

Statistical analysis was undertaken using IBM SPSS Statistics version 25. Linear regression analysis was used for between-group analysis (controlling for age and sex), with post-hoc comparisons. Participants with mean confidence ratings over 5.5 (of a maximum possible rating of 6), and/or very skewed meta-d’/d’ results (defined as over 2 or under-2) were excluded. Alternative tests were used where data did not meet assumptions for parametric data.

### 2.4. Research Ethics

All participants provided informed written consent. Ethical approval was given by the South West-Cornwall and Plymouth Research Ethics Committee, REC reference 15/SW/0298 and IRAS project ID:188539. The study was funded by the BRACE charity.

## 3. Results

### 3.1. Participant Characteristics

A total of 21 people with FCD, 17 with nMCI, and 25 HC participated in the main study (for details see [14]). Of these, the metacognition tasks were completed by 20 people with FCD, 14 with nMCI, and 23 HC. The demographic and global cognitive performance findings for participant groups who completed the metacognitive tasks are shown in Table 1.

### 3.2. Memory Task Results

Accuracy and confidence on the memory task is shown in Table 2.

There was a significant effect of group on mean accuracy (linear regression with age and sex as co-variates, *p =* 0.02, R 0.410, R square 0.168). Mean accuracy over all trials differed between FCD and HC groups (one-way ANCOVA, age and sex as co-variates, F(2, 57) = 4.48, *p =* 0.003). No other significant between group differences were found.

There was a trend for increasing study time to lead to greater accuracy, but this was not significant after controlling for age and sex, in either the cohort as a whole or at a group level (repeated measures ANOVA, Greenhouse-Geisser cohort F(1.81, 95.78) = 2.88, *p =* 0.061, FCD F(1.81, 28.95) = 1.35, *p =* 0.27, nMCI F(1.72, 18.90) = 6.4, *p =* 0.52, HC F(1.79, 35.69) = 0.49, *p =* 0.60.). There were no significant between-group differences on accuracy over 30 or 90 s of study time (linear regression analysis, co-variates of age and sex; 30 s *p* = 0.086, 90 s *p* = 0.133). Over the 60 s study time window, there was a significant effect of group (linear regression, co-variates of age and sex R 0.431, R square 0.186, *p =* 0.012). In one-way ANCOVA (F(2, 52) = 3.68, *p =* 0.032), these differences were driven by significant differences between the FCD and HC groups (*p =* 0.028), and between the nMCI and HC groups (*p =* 0.031); there was no significant difference in accuracy between the FCD and nMCI groups.

No significant between-group differences on confidence were found (linear regression with age and sex co-variates: *p =* 0.69; mean confidence F(3, 53) = 0.98, *p =* 0.41; 30 s *F*(3, 53) = 2.23, *p =* 0.095; 60 s *F*(3, 53) = 0.75, *p =* 0.64; 90 s *F*(3, 25) = 0.49).

Confidence levels were overall higher when greater study time of the stimulus was allowed (repeated measures ANOVA, F(1.45, 78.83) = 22.47, *p* < 0.005). At a group level, confidence was higher with longer study time in the FCD group (repeated measures ANOVA, Greenhouse-Geisser F(1.58, 25.32) = 6.69, *p =* 0.007); pairwise comparisons: 30 s vs. 60 s *p =* 0.046, 60 s vs. 90 s n.s., 30 s vs. 90 s *p =* 0.012). In the nMCI and HC groups, confidence did not significantly increase with longer study time (nMCI repeated measures ANOVA, Greenhouse-Geisser F(1.17, 12.81) = 0.46, *p =* 0.537), HC F(1.59, 31.86) = 4.0, *p =* 0.63).

**Figure 1:**
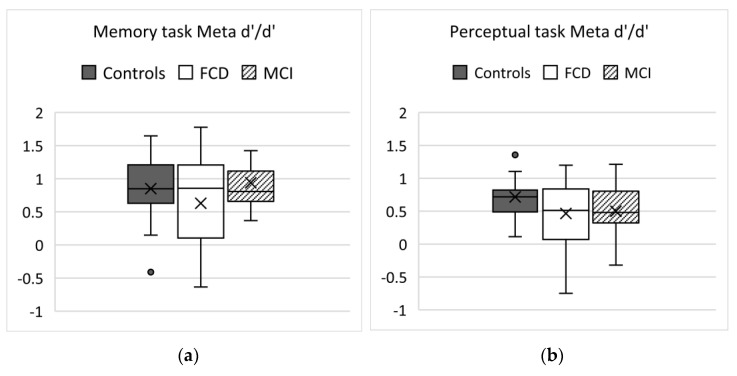
(**a**) Meta-d’/d’ group results on the memory task (all trials). (**b**) Meta-d’/d’ group results on the Perception task.

Memory task Meta-d’/d’ (shown in Figure 1a) was not significantly different between the groups (linear regression analysis controlling for age and sex; F(3, 47) = 0.861, *p =* 0.47). Figure 1 shows Meta d’/d’ across both tasks—note that under ideal circumstances Meta d’/d’ is 1. Meta d’/d’ is calculated using all trials in a task, therefore we cannot comment on how study time in the memory task impacted on metacognition.

The median is shown with the central line, and mean marked X. Outliers above 2 or below-2 excluded (1 participant from the FCD group, 4 HC and 6 nMCI participants). Participants with mean confidence ratings over 5.5 were excluded (2 FCD, 1 HC, and 2 nMCI). Under ideal conditions, Meta d’/d’ is 1.

### 3.3. Perceptual Task Results

Results of the perceptual task are shown in Table 3. Those with very high confidence ratings (mean of over 5.5 out of 6) were excluded from the analysis (1 FCD, 1 HC, 2 nMCI). There were no differences in between-group performance after controlling for age and sex (one-way ANOVA, p 0.40) or confidence (one-way ANOVA, p 0.44) or Meta-d’/d’ (p 0.25; Figure 1b).

Participants with very high confidence ratings (over 5.5 out of 6) were excluded (2 participants from the FCD group, 1 from HC group, and 2 from the nMCI group).

### 3.4. Domain General Index

**Figure 2:**
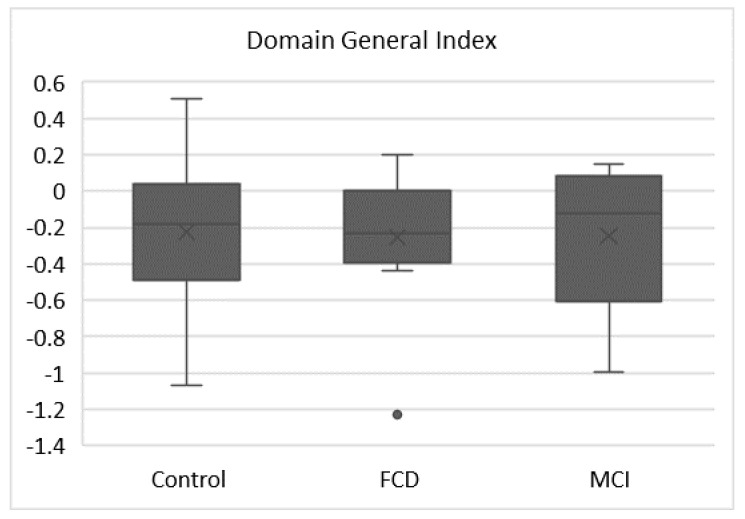
Domain General Index. The DGI is calculated as: [log (Meta-d’/d’)Perceptual] minus [log(Meta-d’/d’)Memory].

The DGI is based on the difference between Log Meta-d’/d’ on the perceptual and memory tasks. A deviation from zero indicates a difference in metacognitive efficacy between the two tasks. The DGI was calculated for 20 HC, 11 people with FCD, and 9 with nMCI (those participants with valid results on both tasks). As can be seen in Figure 2, all groups showed higher metacognitive performance on the memory task, giving a negative value for the DGI (mean values for HC -0.22, FCD -0.26, nMCI -0.27). The difference between perceptual and memory task metacognition was significant in the HC (Wilcoxon Signed Rank test, *p =* 0.02) and FCD groups (Wilcoxon Signed Rank test, *p =* 0.026), but not the nMCI group (*p =* 0.139). There were no significant between-group differences on the DGI (Kruskall–Wallis test *p =* 0.952).

## 4. Discussion

Functional Cognitive Disorder is a little understood but very common cause of cognitive symptomatology, particularly in midlife adults [5,18]. Here, we explored mnestic and perceptual metacognition in well characterised groups of mid to older life adults with FCD, nMCI, and normal cognition [14]. Our primary hypothesis was that the FCD group would show impaired mnestic metacognitive efficacy, with a tendency to under-rate their performance. However, no metacognitive differences emerged between the groups. This was in the context of weaker performance by the FCD and nMCI groups on the memory task. The primary difficulty experienced by the FCD and nMCI groups was in completing the task itself, rather than evaluating their performance.

The FCD group gained significantly in confidence as the study time increased, despite no significant improvement in performance. The FCD group appeared to show a dissociation between confidence and performance. Metacognitive efficacy was calculated using all trials, therefore we cannot comment on whether differences occurred over the three study time windows. This study was designed to detect differences in metacognition, rather than in performance and confidence. It would be of interest to specifically explore confidence in people with FCD—it is possible that differences in self-confidence may be a significant driver of self-reported cognitive symptoms.

Metacognitive efficacy was lower for the perceptual task than the memory task in the HC and FCD groups. This supports the idea that metacognition is domain (or object) specific; that is, one’s self-analysis is specific to the object under scrutiny [1]. An individual may be highly insightful into one cognitive domain whilst having limited awareness of their functioning in another [19]. This point is of high relevance to both metacognitive researchers and clinicians. When targeting research tasks, it is key to specify the object of interest. The object most affected may vary between different diseases, for example in behavioural variant Frontotemporal Dementia function and awareness of social cognition is particularly severely affected [20]. The tasks used in the present study have previously been administered to people with focal brain lesions [16]. People with lesions to the anterior prefrontal cortex showed impaired perceptual metacognition despite intact task performance and mnestic metacognition. This was in contrast to a group with temporal lobe lesions, who retained intact metacognition for both perception and memory. The anterior prefrontal cortex is heavily implicated in metacognitive processing, with evidence from lesioning and tractography studies suggesting that prospective metamemory is subserved by the medial prefrontal cortex, and retrospective metamemory by lateral regions [21]. However, it should be pointed out that the perceptual task using a staircasing procedure to maintain a consistent performance level over multiple trials. It is always difficult to develop exactly equivalent tasks targeting different cognitive domains, and inherent differences in task style could be responsible for apparent domain specific differences in metacognition.

How very early stage symptomatic neurodegeneration impacts on metacognition is debatable. Conflicting evidence is seen across different studies, with some finding people with amnestic MCI to have intact metacognition [22], and others identifying significant deficits [23]. Vannini et al. identified correlations between metacognition and FDG-PET activity in the posterior cingulate and hippocampal cortices in people with amnestic MCI, coupled with reduced functional connectivity between the posterior cingulum, orbitofrontal cortex, and the inferior parietal lobes [13]. The spectrum of MCI is wide, and the definition variable. Most studies (including that presently reported) do not have a biomarker-based diagnosis, therefore are vulnerable to the inclusion of a mixed population, some of whom will have no neuropathology, whilst others are in the early stages of symptomatic Alzheimer’s disease. Whilst we endeavoured to select patients who had additional evidence of a neurodegenerative aetiology for their MCI, as we lacked biomarkers in the form of spinal fluid or amyloid or tau PET analysis, we cannot with certainty classify the underlying disease processes present. The presence of either persons with normal brain structure, or very limited neurodegenerative changes could explain why the nMCI group did not show any metacognitive deficits in the present study.

An interesting point arising from the wider FND literature is the possibility of symptoms being influenced by lay beliefs. In functional tremor, restraint of the affected limb may result in the tremor spreading to other body parts, a phenomenon at odds with the neurobiology of the motor system [24]. A tubular visual field defect can be demonstrated in some people with functional visual loss, where the diameter of the defect does not change with distance from the examiner, against optical laws [25]. Abnormal attention is implicated in functional movement disorders, coupled with abnormal beliefs and expectations, and an abnormal sense of agency [24]. A potential explanation for FCD is that there is excessive attention towards minor memory lapses, which are actually within the normal range of human everyday cognition: walking into a room and not recalling why one is there; experiencing a mnestic block for a noun, then recalling it later. Abnormal attention towards these events and an interpretation of these as being significant errors may represent exaggerated lay beliefs about cognition. Individuals with symptomatic neurodegeneration typically struggle to recount their cognitive errors, whilst those with FCD can give a fluent and detailed history [26]. Failure to recognise that excellent personal narrative ability demonstrates good retrospective memory functioning is a marker of FCD. There are diagnostic pitfalls for the unwary, particularly individuals with very mild neurodegeneration who may also give a rich history of their symptoms, and those with more significant symptoms but with a good social façade and convincing confabulation—the latter can often be identified by their informant’s reaction during the consultation. This theory of misapplied attention in FCD coupled with abnormal beliefs about cognitive abilities (typically memory) would explain the deficits on performance seen during the memory task and intact metacognition. Altered attention and an expectation of failure could conspire together to impair word list encoding, resulting in impaired performance. Future work evaluating how FCD impacts on attention would help investigate this hypothesis, alongside an exploration of beliefs about cognition held by lay people.

Limitations of this work include relatively small group numbers, although these are not uncommon in the literature. Diagnosis of FCD can be clinically challenging, and there is no definitive tests for this condition, which will contribute to group heterogeneity and raise the possibility of misdiagnosis of neurodegeneration. As previously discussed, nMCI participants did not have neurodegenerative biomarkers available; therefore, this group will also be heterogeneous. Diagnoses in both the FCD and nMCI were made after robust clinical assessment and expert discussion, but cannot be clinically ‘definite’ due to limitations of diagnostic definitions and available clinical tests. The terminology and definitions used for FCD are still in a state of flux, but we would hope that consensus will soon emerge and this will greatly facilitate future studies of the neural basis of FCD. Finally, participants gave feedback that they found both the tasks challenging. A more naturalistic task design evaluating metacognition during everyday cognitive tasks may be more sensitive to change.

In conclusion, this study did not find metacognitive deficits in in groups of well characterised participants with FCD and nMCI. Performance on a memory task was impaired in both groups, but perceptual ability was preserved. A dissociation between perceptual and mnestic metacognition was observed, supporting the hypothesis that metacognition is domain specific.

## Figures and Tables

**Table 1:** Group demographics and cognitive functioning (mean values; significant *p* values in bold).

	FCD	nMCI	HC	FCD vs. HC *p* Value	FCD vs. nMCI *p* Value	nMCI vs. HC *p* Value
Female:Male	9:11	8:6	16:7		0.043	
Age (years)	57.2	70.6	60.7	0.882	0.003	0.048
Years of Education	13.9	14.4	15.1	0.48 *		
MoCA	23.7	23.6	27.7	0.003	1.00	0.001

* No significant difference across samples found; therefore, multiple comparisons not performed. Sex: Pearson’s Chi-square. df = 2, Chi-square value 6.28, *p* = 0.043 (adjusted residual significant for HC only: male-2.3, female 2.3). Age: Kruskal–Wallis with Bonferroni correction. Years of education: one-way ANOVA. = 0.75, df = 56, 2, *p* = 0.48. MoCA: Kruskal–Wallis with Bonferroni correction.

**Table 2:** Memory Task Performance and Confidence.

	Study Time	% Correct (Mean)	% Correct (SD)	Confidence ^1^ (Mean)	Confidence (SD)	Meta-d’/d’	Meta-d’/d’ (SD)
FCD	30 s	60.00	9.43	2.84	0.82	0.63	0.68
	60 s	66.10	8.96	3.23	0.78		
	90 s	64.95	13.60	3.57	0.93		
	Mean	63.65	7.75	3.19	0.74		
nMCI	30 s	62.14	7.98	3.37	0.60	0.94	0.47
	60 s	62.86	8.25	3.63	0.50		
	90 s	66.86	10.58	3.77	0.64		
	Mean	63.96	9.04	3.59	0.59		
HC	30 s	66.87	8.98	3.41	0.75	0.85	0.46
	60 s	71.83	8.16	3.70	0.73		
	90 s	74.96	11.00	4.01	0.60		
	Mean	71.22	7.19	3.71	0.61		

^1^ Confidence ratings were from 1.00 to 6.00. Note only one value to Meta-d’/d’ is given per participant, using performance over all trials.

**Table 3:** Performance accuracy, confidence and Meta-d’/d’ on the perceptual task.

	% Correct (Mean)	% Correct (SD)	Confidence (Mean)	Confidence (SD)	Meta-d’/d’	Meta-d’/d’ (SD)
FCD	63.97	5.55	3.71	0.51	0.50	0.51
nMCI	64.50	2.10	3.43	0.60	0.51	0.40
HC	65.86	1.85	3.88	0.69	0.72	0.32

## Data Availability

Limited, group level data may be available subject to ethical approval.
